# Atopic eczema is an environmental disease 

**DOI:** 10.5414/ALX02258E

**Published:** 2021-08-23

**Authors:** Daria Luschkova, Katharina Zeiser, Alika Ludwig, Claudia Traidl-Hoffmann

**Affiliations:** 1 Department of Environmental Medicine, Faculty of Medicine, University of Augsburg, Augsburg,; 2 Professorship of Sociology with a focus on health research, Faculty of Philosophy and Social Sciences, University of Augsburg, Augsburg, Germany and; 3 Christine Kühne-Center for Allergy Research and Education (CK-CARE), Davos Wolfgang, Switzerland

**Keywords:** atopic eczema, neurodermatitis, environmental factors, hygiene hypothesis, air pollut- ants, nutrition, psychosocial factors, biodiversity, microbiome dysbiosis, S. aureus, pollen, allergenicity, ambrosia, climate change, climate resilience

## Abstract

It is obvious that social, biogenic, and anthropogenic environmental factors, as well as nutrition contribute to the development and course of atopic eczema. Social deprivation and stress have a negative impact on atopic eczema symptoms, and social change in recent decades has led to a “westernized” lifestyle associated with high prevalence of atopic eczema in industrialized countries. Urbanization leads to an increase in air pollution and a decrease in biodiversity, which negatively affects atopic eczema. Climate change alters the allergenicity of pollen, which increases atopic eczema symptoms in some patients during the pollen season. Protective natural and social factors for the prevention of atopic eczema and for the promotion of “climate resilience” should be given greater consideration in future research.

## Introduction 

Both hereditary predisposition and the environment of a person shape their health. Some disease patterns are exclusively caused by environmental factors, others, such as atopic eczema, are at least favored, triggered, or maintained by environmental factors. Atopic eczema (AE) (synonyms: neurodermatitis, atopic dermatitis) is one of the most common chronic diseases worldwide. Approximately 2.5 – 3.5% of adults and 10 – 15% of children in western industrialized countries are affected [1]. AE also plays a special role in the development of other allergic diseases. The epidermal barrier disorder makes the skin more permeable to allergens and trigger factors, and promotes sensitization. Hay fever, asthma, and manifest allergies usually develop later in the course of the “atopic march”. 

In the 19^th^ century, it was assumed that the disease was caused by an inflammation of the nerves. The term “neurodermatitis” is derived from this. After that, for a long time a genetic predisposition was considered the only reason for the development of the disease. Although the genetic component plays a role in the predisposition to dry skin and atopy, there is also evidence that a variety of environmental factors influence AE. The increase in the prevalence of AE, as well as other atopic diseases, in recent decades in most industrialized countries suggests that the causes are environmental changes rather than genetic factors. 

By studying environmental factors, the disease pattern can be better understood, and based on this, preventive measures can be developed. This article provides an overview of environmental factors associated with atopic eczema. 

## What is environment? 

**Figure 1.**
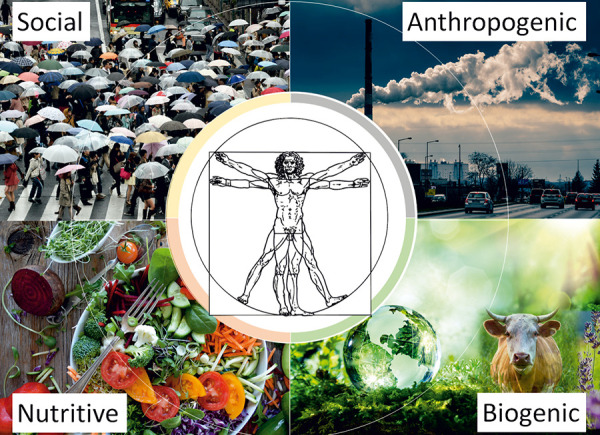
Various environmental factors that influence each other.

Various definitions exist for the term environment. Environment can be defined narrowly as the natural world surrounding humans or more broadly as all environmental factors affecting a living being. In this article, the term environment is broadly defined to include (psycho)social, anthropogenic, nutritive, and biogenic aspects (Figure 1). 

## Social environment 

**Figure 2.**
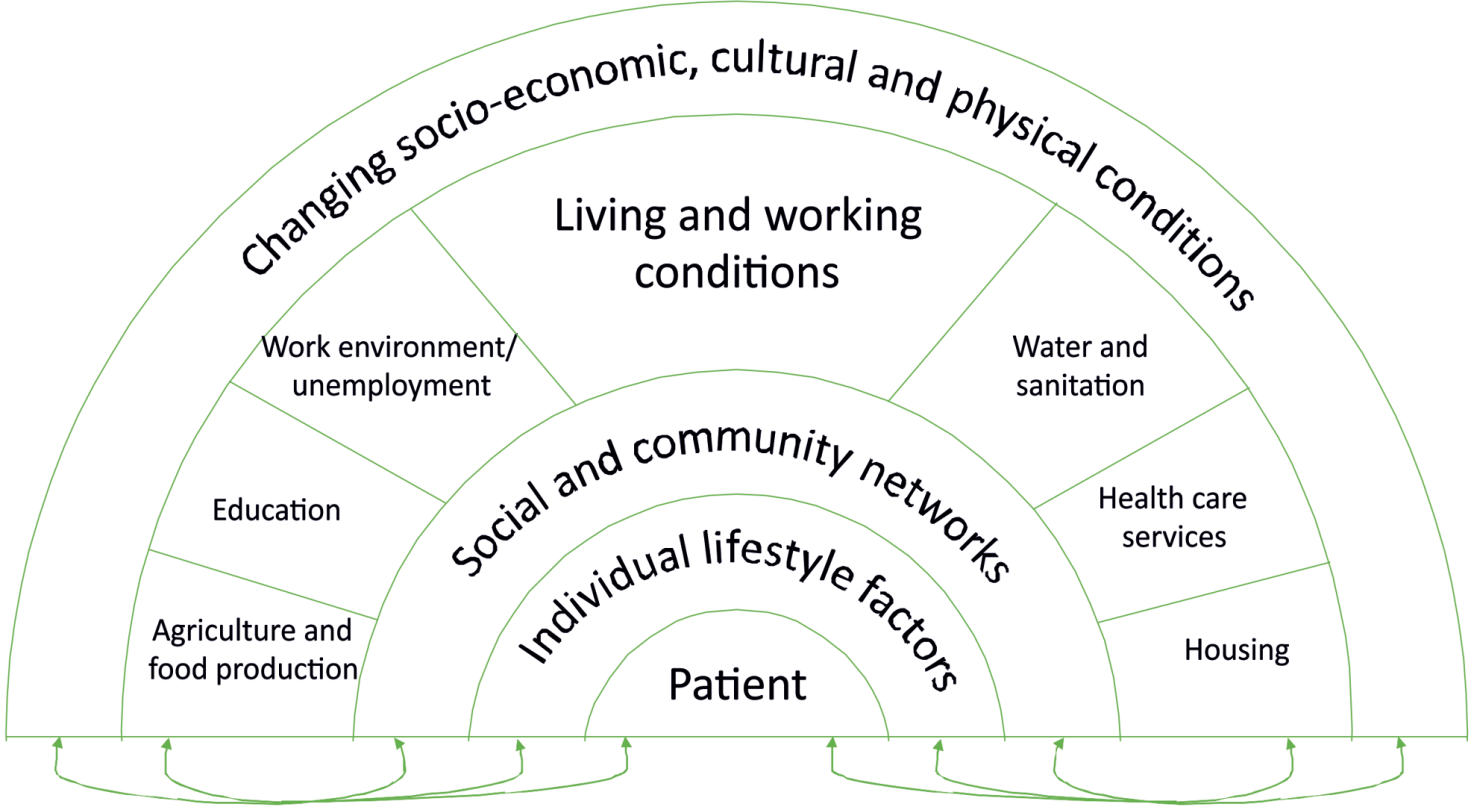
Social environmental factors related to atopic dermatitis, rainbow model. Modified according to [2].

With regard to the social environment, there are numerous risk and protective factors that interact with each other and can impair or promote health (Figure 2). 

For example, a social gradient has been shown in symptom severity, meaning that people from lower socioeconomic status groups tend to have more severe symptoms. This is also true for AE, although the prevalence tends to correspond to an inverse social gradient, i.e., the condition is more common among high social status [3]. Structural racism is also associated with symptom severity. For example, a study from the USA showed that African American children were more often socioeconomically disadvantaged and had more severe AE symptoms [4]. 

As society changes, sociologists are observing an increase in perceived stress levels in addition to increasing urbanization, which is leading to an increase in air pollutants, among other things. At the same time, prenatal stress has been shown to increase the risk of AE [5], and AE patients often report that stress triggers or exacerbates their symptoms [6]. Social norms of beauty and health, which lead to experiences of stigmatization when skin conditions are visible, can trigger additional stress and psychological sequelae, creating a kind of “vicious cycle.” In addition, health is increasingly defined as a problem that those affected must solve themselves. This can lead to additional feelings of guilt for those affected with AE if they do not succeed. 

Other social developments can have not only a negative but also a beneficial effect on AE. One example is digitalization, which may not only lead to “digital stress” but has also contributed to the development of atopic eczema apps that help patients document their symptoms and their triggers. These apps can be helpful in better managing the disease. 

Healthcare, and specifically the design of reimbursement, also plays a key role in symptom severity. AE is associated with significant costs for patients [7]. For example, in order to relieve patients from precarious socioeconomic backgrounds in particular, there have been calls for several years for health insurance companies to reimburse basic therapy. Also other measures, which were evaluated as helpful in the last AWMF guideline “Atopic eczema” (for example relaxation training or behavior therapy with appropriate indication) are not promoted and used sufficiently so far [8]. Although about three quarters of all affected people state that their symptoms are stress-related or psychologically caused, only about one quarter of the affected persons receive psychotherapeutic support [1]. 

Conflicts in the family, partnership, or workplace can also trigger stress, which can exacerbate AE symptoms. However, the social environment can also be beneficial for people with AE. Here, further research is needed to find out to what extent social support or social networks might have a positive influence on AE [6]. 

## Diversity of the environment as a protective factor 

There is no question that people living in industrialized countries and especially in cities are less exposed to diverse microorganisms such as viruses, bacteria, fungi, and parasites. The “jungle” or “hygiene” hypothesis explains the increase in atopic diseases by the decrease in immune-stimulating factors. 

This is supported by observations among the traditionally living Amish and Hutterites in the USA. Both communities are agricultural. In contrast to the Amish, however, the Hutterites work with modern machinery. Children of the Amish show a lower allergy risk than children of the Hutterites. Amish house dust has been found to contain higher levels of bacterial components that are protective of the immune system [9]. It is also believed that spending time in animal barns, especially cattle stables, and exposure to stable dust components (β-lactoglobulin) protect against allergic diseases (“farm effect”) [10]. 

The newly emerged “biodiversity hypothesis” extends the hygiene hypothesis. It states that contact with nature enriches the human microbiome, boosts the immune system, and thus protects against allergies and inflammatory diseases [11]. Biodiversity is the variety of all living organisms and their interdependencies on earth. Passive (environment of residence) and active (lifestyle, hobbies, animal contacts) contact with environmental microorganisms plays a significant role in the development of allergies. Finnish researchers have shown that adolescents who lived immediately next to a species-rich vegetation and land use type were less likely to have atopy [12]. Families with many children or a dog in the household may also have a protective effect. 

**Figure 3.**
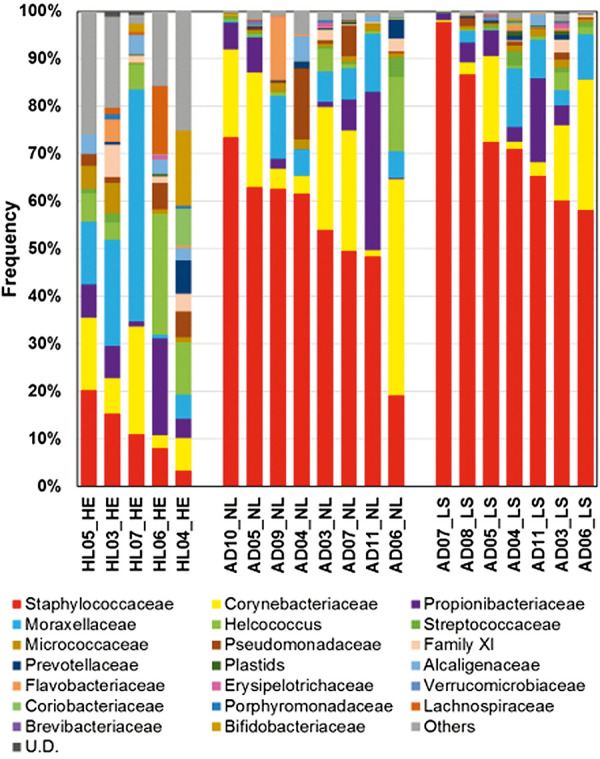
Microbiome analysis in healthy individuals and in AE patients. In healthy individuals (HE) the skin microbiome is colonized by different bacterial species, in AE patients (AD) the diversity of the microbiome decreases. Dysbiosis of the skin flora is particularly pronounced in lesional skin (LS). Modified from [13].

Biodiversity and functioning ecosystems are an important foundation for our health, food, and resources. From an ecological perspective, the human body is a habitat for a broad spectrum of microorganisms – the microbiome. It orchestrates the interaction between our cells and the environment. AE patients are known to have dysbiosis of the skin microbiome. Lesional skin and decreased diversity of the microbiome go hand in hand and are accompanied by excessive growth of the pathogenic *Staphylococcus aureus* (*S. aureus*, Figure 3). This pathogen is in turn associated with worsening of the skin appearance and disease severity in patients [13]. In particular, the first months of life appear to be a “critical window”. Studies show that colonization of the skin with commensal, i.e., beneficial, staphylococci within the first months after birth reduces the risk of later developing AE [14]. In contrast, infants colonized with pathogenic *S. aureus* showed an increased risk of developing AE [15]. 

A diverse gut microbiome also appears to have a beneficial effect. For example, it has been shown that infants who developed AE had lower numbers of Bifidobacteria in stool samples [16]. Breast milk and the administration of probiotics have been reported to support the development of a healthy gut microbiome and even reverse the negative effects of antibiotic treatment (for example, in cesarean births) on newborns [17]. In addition, a diverse diet appears to protect against allergies, especially in at-risk infants in their first years of life. 

Another example is increased hygiene through detergents. They may affect the microbiome and the protective function of the skin barrier and increase the sensitivity of the skin to trigger substances in AE. Hand hygiene has increased, particularly in the context of the current COVID-19 pandemic. Further protective measures such as distance regulations, reduction of social contacts, and travel barriers could also influence the human microbiome on a global scale [18]. 

## Outdoor and indoor anthropogenic air pollutants 

Directive 2008/50/EC on ambient air quality and cleaner air in Europe sets limits for carbon monoxide (CO), sulfur dioxide (SO_2_), nitrogen dioxide (NO_2_), benzene, and particulate matter (PM10, PM 2.5) emissions. The emissions are largely caused by the combustion of fossil fuels. Children growing up near busy roads show an increased risk of developing atopic diseases. In addition, air pollutants can increase the severity of manifested AE in children [19]. Pollutants (for example, particulate matter) can penetrate to the deeper layers of the skin and compromise the epidermal barrier. Proinflammatory reactions, oxidative stress, and epigenetic imprinting are also being explored as possible mechanisms of action. 

Exposure to volatile organic compounds (VOCs) in indoor environments, for example from building materials and furnishings, shows similar effects. Pollutant concentrations below the respective thresholds can already trigger symptoms [20]. This is significant insofar as we increasingly spend more time indoors. In addition, renovation work, painting of walls, and new furniture increase the risk of AE, especially in newborns [21]. Another study proved that a combination effect of VOCs and allergens from house dust mites may even increase skin barrier damage in those AE patients sensitized to house dust mites [22]. However, the most important allergenic factor indoors is tobacco smoke. Tobacco smoke has a significantly greater effect on children of atopic parents than on children of non-atopic parents. Thus, there seems to be a correlation between genetic susceptibility and exposure to pollutants [23]. 

## Climate change and biotic factors 

Anthropogenic air pollutants are also the most important cause of global warming, i.e., climate change. AE symptom severity is associated with seasonal variations. In children, weather often triggers exacerbations in fall and winter, and in adults, more often in summer. Heat and lack of night-time air cooling may also exacerbate itching and sleep loss. Extreme weather could therefore worsen the clinical picture. 

**Figure 4.**
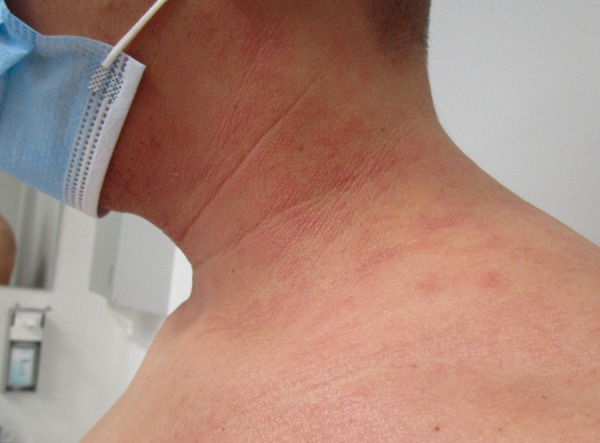
During the pollen season, the skin condition may worsen, especially for eczema of the head and neck area, in aeroallergen-sensitized AE patients. University outpatient clinic for environmental medicine, Augsburg University Hospital.

In addition, air pollution and climate change are affecting the world’s flora and fauna. In particular, wind-pollinated plants and their pollen are a key factor in the development of allergic diseases. 80% of adults suffering from AE are sensitized by specific IgE antibodies to inhalant or food allergens. Several studies have been published demonstrating an association between AE severity and sensitization. There is seasonal variability: in summer and especially on days with high pollen load, symptoms of sensitized patients may increase. In particular, for eczema of the head and neck, aeroallergen sensitization is often responsible for AE exacerbation during the pollen season (Figure 4) [24]. 

**Figure 5.**
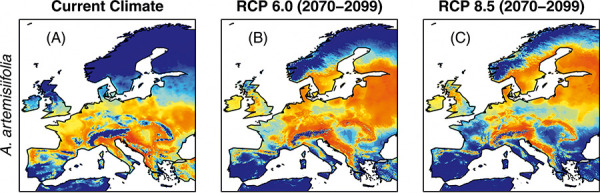
Habitat suitability of *Ambrosia artemisiifolia* in Europe under current climate conditions and the future IPCC climate scenarios RCP 6.0 and RCP 8.5 for the years 2070 – 2099. Modified from [29], Creative Commons CC-BY 4.0.

It is speculated that climate change has an impact on the onset and duration of pollen flight and pollen concentration. This may affect both the timing of onset and symptom severity in patients. Phenological characteristics of plants are very sensitive to changes in environmental factors, especially temperature variability [25]. Higher pollen production and earlier onset of the pollen season have been demonstrated in warmer years and in locations with elevated temperatures, such as urban areas. Air pollutants act as additional stressors on plants. They may respond with changes in protein and metabolite profiles of pollen and increase their allergenicity [26]. For example, birch pollen collected in regions with high atmospheric ozone show increased levels of the major allergen Bet v 1 in pollen samples [27]. A global increase in CO_2_, especially in combination with rising air temperature, may alter pollen allergenicity [28]. In addition, changes in environmental factors create new vegetation niches for previously non-native plant species. An example for this is the invasion of *Ambrosia artemisiifolia* (ragweed, Figure 5). Ragweed originates in North America and was brought to Europe with grain supplies. Due to cross-reactivity with mugwort, new sensitization to ragweed pollen is not necessary to cause allergic symptoms. Therefore, the highly allergenic pollen of the late-flowering plant (August to October) prolongs the symptoms of sensitized individuals into the fall. 

Climate change is producing new ecosystems, accelerating biological invasions, and altering vegetation phases globally. Species with great flexibility in appearance and physiology are better able to respond to climate change. How well humans cope will depend in particular on how well vulnerable populations can adapt to global changes. 

## Conclusions 

Atopic eczema is an environmental disease. However, a large number of the environmental factors that trigger or exacerbate AE can be influenced by humans. People with AE represent a vulnerable population burdened by climate change, increasing global warming, air pollution, as well as increasing stress. Urbanization has reduced biodiversity and changed our lifestyle. Our immune systems have yet to adapt to this relatively new situation. Even if a return to traditional rural life is not possible, it is advisable to introduce natural factors into modern city life, for example through urban planning that provides sufficient green spaces. In addition, a diverse microbiome and diet promote health. Monitoring the amount and timing of pollen in the air and correlating it with symptom severity is also important. Overall, research on the protective factors from nature and social environment to prevent atopic eczema should be encouraged. 

## Funding 

None. 

## Conflict of interest 

The authors declare no conflict of interest.
